# Disruption of the bHLH transcription factor Abnormal Tapetum 1 causes male sterility in watermelon

**DOI:** 10.1038/s41438-021-00695-9

**Published:** 2021-12-01

**Authors:** Ruimin Zhang, Jingjing Chang, Jiayue Li, Guangpu Lan, Changqing Xuan, Hao Li, Jianxiang Ma, Yong Zhang, Jianqiang Yang, Shujuan Tian, Li Yuan, Xian Zhang, Chunhua Wei

**Affiliations:** 1 State Key Laboratory of Crop Stress Biology for Arid Areas, College of Horticulture, Northwest A&F University, Yangling, 712100 Shaanxi China; 2 State Key Laboratory of Vegetable Germplasm Innovation, Tianjin Kernel Vegetable Research Institute, Tianjin, 300384 China

**Keywords:** Pollen, Plant breeding, Transgenic plants

## Abstract

Although male sterility has been identified as a useful trait for hybrid vigor utilization and hybrid seed production, its underlying molecular mechanisms in Cucurbitaceae species are still largely unclear. Here, a spontaneous male-sterile watermelon mutant, Se18, was reported to have abnormal tapetum development, which resulted in completely aborted pollen grains. Map-based cloning demonstrated that the causal gene *Citrullus lanatus Abnormal Tapetum 1* (*ClATM1*) encodes a basic helix-loop-helix (bHLH) transcription factor with a 10-bp deletion and produces a truncated protein without the bHLH interaction and functional (BIF) domain in Se18 plants. qRT–PCR and RNA in situ hybridization showed that *ClATM1* is specifically expressed in the tapetum layer and in microsporocytes during stages 6–8a of anther development. The genetic function of *ClATM1* in regulating anther development was verified by CRISPR/Cas9-mediated mutagenesis. Moreover, *ClATM1* was significantly downregulated in the Se18 mutant, displaying a clear dose effect at the transcriptional level. Subsequent dual-luciferase reporter, β-glucuronidase (GUS) activity, and yeast one-hybrid assays indicated that ClATM1 could activate its own transcriptional expression through promoter binding. Collectively, *ClATM1* is the first male sterility gene cloned from watermelon, and its self-regulatory activity provides new insights into the molecular mechanism underlying anther development in plants.

## Introduction

Male sterility, a common phenomenon in flowering plants, is an important breeding tool for hybrid seed production and heterosis utilization^1^, and male-sterile materials are valuable for the study of anther and pollen development, meiosis, and programmed cell death (PCD)^2–4^. According to the hereditary mode of sterility genes, this vital agronomic trait can be further divided into cytoplasmic male sterility (CMS) and genic male sterility (GMS), which are caused by mitochondrial genes coupled with nuclear genes and nuclear genes alone, respectively^5^. GMS can result in stable and complete male sterility^6^, which is correspondingly related to meiotic irregularity, abnormal development of anther walls, aberrant development of pollen walls, spatiotemporal errors in PCD, and abnormal anther dehiscence^7,8^. As the innermost layer of the anther wall, the tapetum is essential for pollen development due to its roles in nutrition, signaling, microspore release, pollen wall synthesis, and pollen coat deposition^9–12^. A defective tapetum generally leads to abnormal pollen, which causes male sterility in plants^13^.

The development and function of the tapetum are regulated by many transcription factors (TFs), including R2R3 myeloblastosis (MYB), plant homeodomain (PHD) fingers, and basic helix-loop-helix (bHLH) proteins^5,10,13–16^. Among these TFs, members of bHLH subfamilies II and III (a + c)1 have been inferred to play conserved roles in regulating tapetum development in angiosperms^17^. Members of bHLH subfamily III (a + c)1, such as Arabidopsis (*Arabidopsis thaliana*) *DYSFUNCTIONAL TAPETUM 1* (*DYT1*)^18^, *ABORTED MICROSPORE* (*AMS*)^19,20^, rice (*Oryza sativa*), *UNDEVELOPED TAPETUM 1* (*UDT1*)^21^, *TAPETUM DEGENERATION RETARDATION* (*TDR*)^12^, maize (*Zea mays*) *MALE STERILITY 32* (*MS32*)^22^, and tomato (*Solanum lycopersicum*) *MALE STERILITY 10*^*35*^ (*MS10*^*35*^)^23^, have been functionally characterized in terms of their involvement in tapetum and pollen development, e.g., *dyt1* mutants displaying enlarged tapetum and degenerated microspores^18,24^; moreover, the dysregulation of *AMS* results in abnormally enlarged and vacuolated tapetum callose walls and premature microspore degeneration^20,25^. Single mutants of some bHLH subfamily II proteins, such as rice *ETERNAL TAPETUM 1* (*EAT1*) and *TDR INTERACTING PROTEIN 2* (*TIP2*)^3,26,27^, maize *MALE STERILITY 23* (*MS23*)^22^, and Medicago (*Medicago truncatula*) *EMPTY ANTHER 1* (*EAN1*)^17^, display complete male sterility. In addition, three bHLH subfamily II TFs in Arabidopsis, *AtbHLH10, AtbHLH89*, and *AtbHLH91*, were shown to have redundant functions, and defective anther phenotypes were observed only in double and triple mutants rather than in single mutants^28^. All these bHLH TFs function in early tapetum development, while some MYB TFs and PHD-figure proteins are important for late tapetum development^13^. For example, the Arabidopsis MYB TF *AtMYB103/AtMS188* is directly regulated by AMS and regulates tapetum development, callose dissolution, and exine formation during anther development^29,30^. *AtMS1* encodes a PHD-finger protein and plays key roles during various postmeiotic stages^31,32^. Although many genes participating in anther development have been identified in model plant species, male sterility genes have rarely been studied in cucurbit crop species.

Watermelon (*Citrullus lanatus* L.) is a globally important cucurbit crop species with obvious heterosis and exhibits high disease and stress resistance, quality, and yield. As a typical monoecious crop species, highly purified hybrid watermelon seeds are mainly produced by hand pollination, requiring a great deal of labor and time. The application of male-sterile lines can sufficiently overcome these obstacles in hybrid seed production in crop plants^2^. To date, although seven watermelon male-sterile mutants have been reported, glabrous male-sterile (*gms*)^33^, male-sterile dwarf (*ms-dw*)^34^, male sterile 1 (*ms-1*)^35^, male sterile 2 (*ms-2*)^36^, male sterile 3 (*ms-3*)^37^, DAH3615-MS^38^, and Se18^39^, the genetic cloning of the functional genes as well as the related underlying mechanisms are still poorly understood. In this study, the spontaneous complete male-sterile watermelon mutant Se18 and its sibling wild-type (WT) line were used for comparative cytological analysis, revealing that the defective tapetum initiating at stages 6–7 was responsible for complete male sterility. Subsequently, the causal gene *ClATM1* (*Abnormal Tapetum 1*), which encodes a bHLH protein, was identified through a map-based cloning strategy, and its function was validated by the CRISPR/Cas9 gene-editing system. Our findings also revealed the self-regulatory ability of *ClATM1*, providing new insights into the molecular mechanisms underlying tapetum development in plants.

## Results

### Se18 is a complete male-sterile mutant

**Fig. 1:**
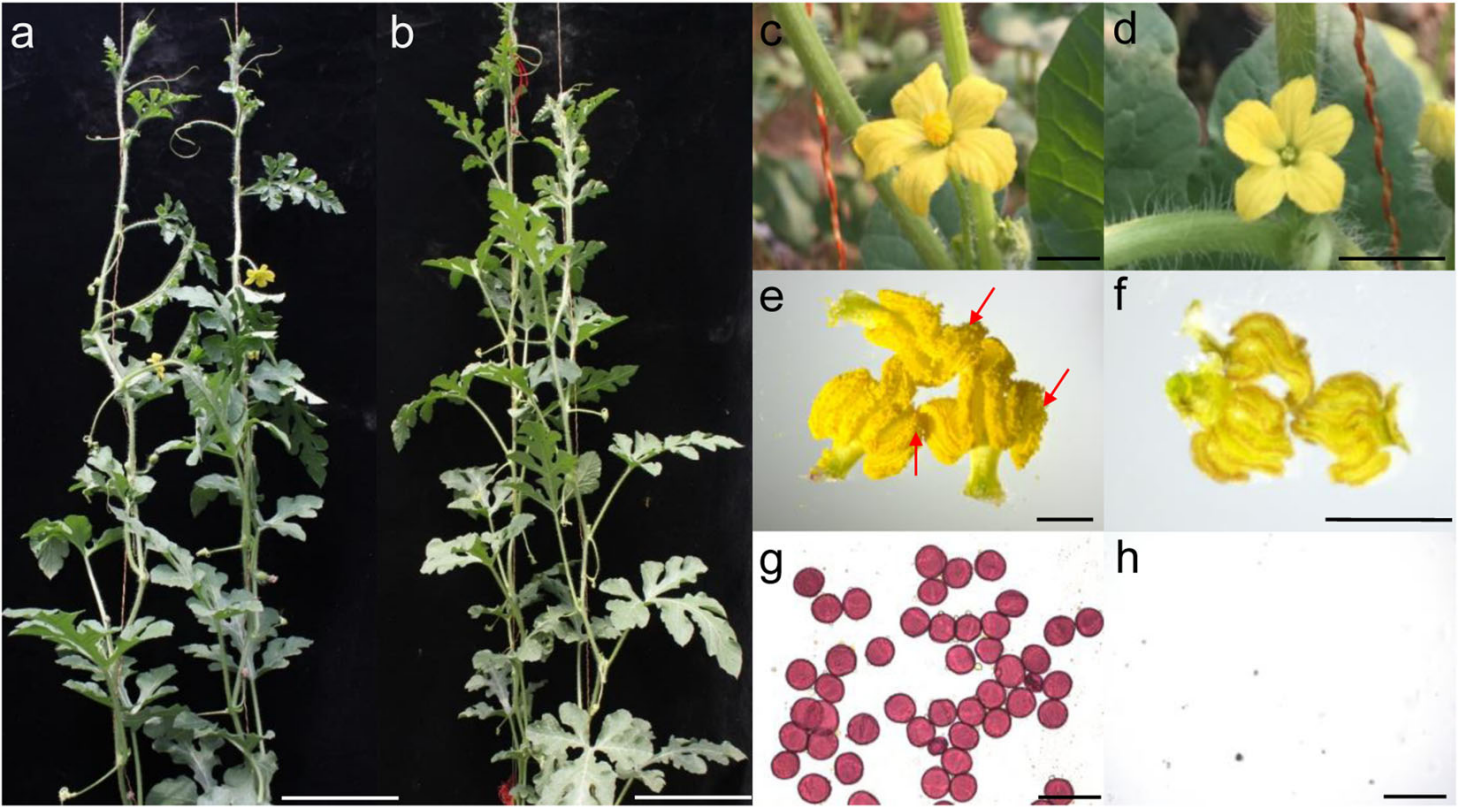
Phenotypic analyses of wild-type (WT) and male-sterile mutant (Se18) plants. **a–b** Vegetative growth of WT (**a**) and Se18 (**b**). Scale bars, 10 cm. **c–d** Male flower phenotypes of WT (**c**) and Se18 (**d**). Scale bars, 2 cm. **e–f** Anther morphological characteristics of the WT **(e)** and Se18 **(f)**. The pollen grains are indicated by red arrows. Scale bars, 2 mm. **g–h** Analysis of pollen viability of the WT **(g)** and Se18 **(h)**. Scale bars, 100 μm

Previously, we identified a spontaneous watermelon mutant, Se18, which is completely male sterile, and the phenotype was stably inherited in the propagules^40^. The Se18 mutant exhibited normal vegetative growth with an indistinguishable morphology compared with that of WT plants (Fig. 1a, b) and produced normal female flower organs^41^. However, compared with the WT, Se18 flowered later and produced male flowers of reduced size and with pale yellow petals and degenerated stamens (Fig. 1c, d). Moreover, visible pollen grains had obviously formed in the WT (Fig. 1e), which, upon further staining by Alexander solution, were round, full, and deep red (Fig. 1g). In contrast, no pollen grains were observed on the anther surface of opening flowers in Se18 (Fig. 1f), as evidenced by the results of the pollen staining assay (Fig. 1h).

### Abnormal tapetum and persistent callose are observed in Se18 anthers

To further explore the cytological defects during anther development, transverse sections of WT and Se18 anthers of different sizes were examined in both the WT and Se18. On the basis of the anther cytological patterns of Arabidopsis^42^ and rice^43^, the watermelon anther developmental process was divided into several stages in WT plants according to the transverse diameter of male floral buds collected in 2018, 2019, and 2020: stage 5, 1.5–2.0 mm; stages 6–7, 2.0–2.5 mm; stage 8a, 2.5–3.0 mm; stage 8b, 3.0–3.5 mm; stage 9, 3.5–4.0 mm; and stage 10, 4.0–4.5 mm (Table S1).

**Fig. 2:**
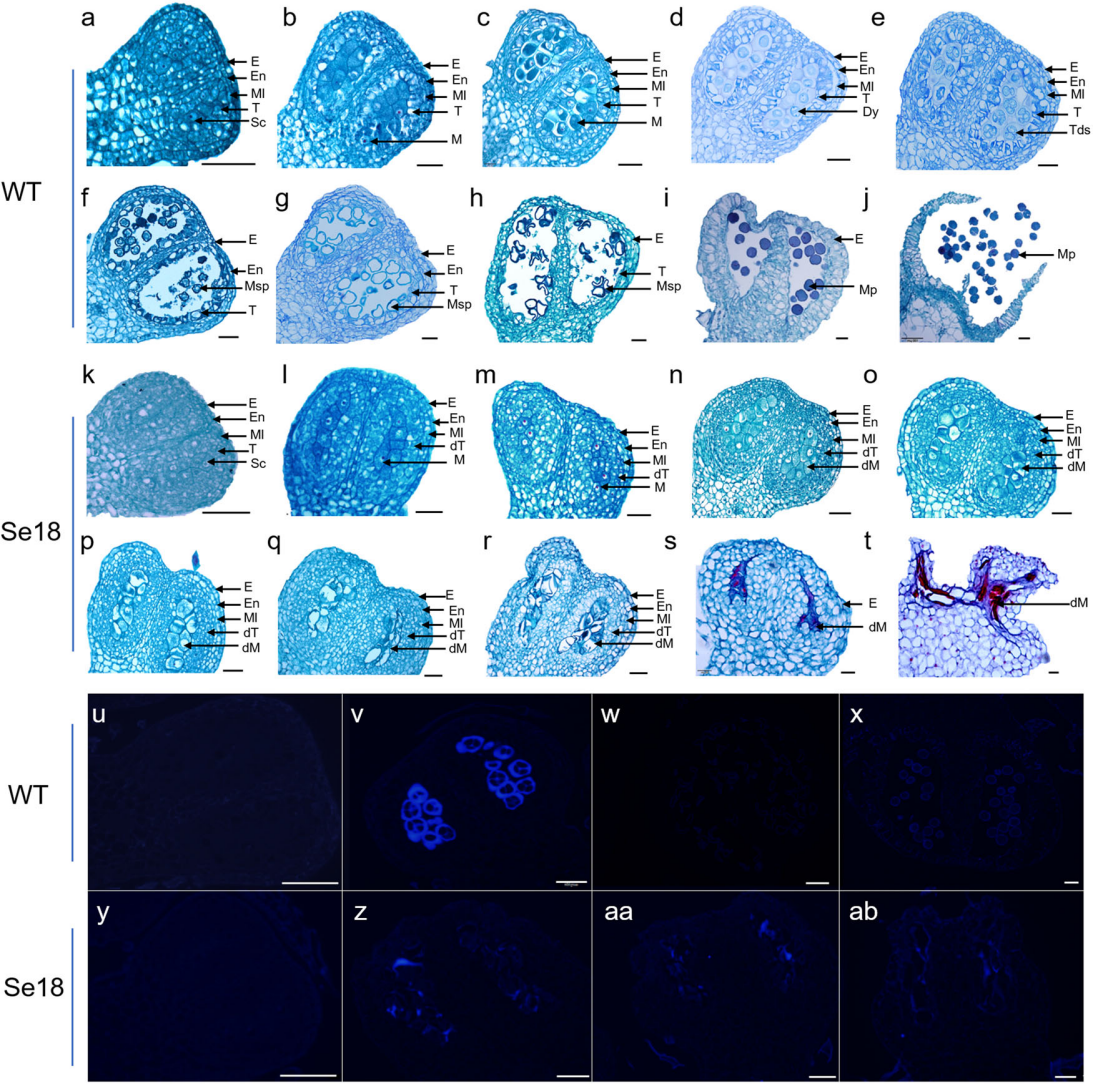
Comparison of anther and pollen development in the WT and Se18 mutant. **a–j** Transverse sections of WT anthers at stages 5–12 and 14. **k–t** Transverse sections of Se18 anthers of different sizes. **u–x** Callose analysis of the WT at the premeiotic (**u**), tetrad (**v**), posttetrad (**w**), and pollen grain (**x**) stages. **y–ab** Callose analysis of Se18 anthers of different sizes. The corresponding sizes of the floral bud diameters (mm) are presented in Table S1. dM defective microsporocyte, dT defective tapetum, Dy dyad cell, E epidermis, En endothecium, M microsporocyte, Ml middle layer, Mp mature pollen, Msp microspore, Sc sporogenic cell, T tapetum, Tds tetrads. Scale bars, 50 μm

For WT plants, sporogenic cells (Sc) and four-layer anther walls (the epidermis (E), endothecium layer (En), middle layer (Ml), and tapetum (T), from the outside to the inside) were visible at stage 5 (Fig. 2a). The Sc underwent several divisions to form microsporocytes (Ms) at stage 6 (Fig. 2b), which initiated meiotic division at stage 7 (Fig. 2c). Moreover, the functional tapetal cells were nearly rectangular in shape and regularly surrounded Ms (Fig. 2b–e). Subsequently, Ms underwent meiosis I to form dyads (Dys) with cell plates at stage 8a (Fig. 2d). Tetrads (Tds) of four haploid microspores were formed after meiosis II at the end of stage 8b, which was accompanied by vacuolation and degeneration of the tapetal cells (Fig. 2e). Individual microspores (Msp) were released along with the continued degradation of tapetal cells at stage 9 (Fig. 2f). Then, Msp underwent mitosis, formed exine walls, and developed into mature pollen (Mp) during stages 10–12 (Fig. 2g–i), followed by their release from dehiscent anthers at stage 14 (Fig. 2j).

There were no obvious cytological differences in the formation of Scs and four-layer anther somatic cells between the WT and Se18 plants (Fig. 2a, k). However, the anther developmental process of Se18, which lacked functional tapetal cells, dyads, tetrads, microspores, and pollen grains at each stage (Fig. 2l–t), was different from that of WT. The defective tapetum (dT) was slightly distinct and initially had an atypical shape (Fig. 2l, m). Subsequently, the dT proliferated and generated a multilayered tapetum (smaller in size, irregular in shape, and not radially elongated), which compressed the anther locule, and no typical dyads or tetrads were detected in Se18 anthers (Fig. 2n–r). Afterward, tapetal cells became highly swollen and vacuolated spontaneously; consequently, the defective microsporocytes (dMs) were crushed and degraded (Fig. 2s). Finally, the anther wall and the dMs completely disintegrated (Fig. 2t).

Callose (β-1,3-glucan) is vital for both the formation and release of microspores, and the tapetum can produce β-1,3-glucanase for degradation^44,45^. There was no callose in either WT or Se18 anthers at the premeiotic stage (Fig. 2u, y). At the tetrad stage, a thick callose wall was clearly present surrounding the tetrad in the WT anthers (Fig. 2v), which was diminished after the tetrad stage to release the microspores (Fig. 2w). Subsequently, autofluorescence of the exine wall of developing pollen was observed at the pollen grain stage (Fig. 2x). In contrast, the Se18 anther callose walls were visible but were obviously thinner compared with WT in the early stage (Fig. 2z); they remained detectable but were scattered and irregularly shaped in the following stages (Fig. 2aa–ab), suggesting that callose degeneration is defective in the Se18 mutant. These results demonstrated that the male sterility of Se18 resulted from abnormal tapetum development.

**Table 1:** Genetic inheritance analysis of different generations of Se18, M08, and WT plants

Generation	Total	Male fertile	Male sterile	Expected segregation	*χ*^2^ value	*P* value
WT	30	30	0			
Se18	30	0	30			
F_1_Se18×WT	50	50	0			
F_2_Se18×WT	355	264	91	3:1	0.076	0.783
Se18×(Se18×WT), BC1	157	81	76	1:1	0.159	0.69
M08	30	30	0			
F_1_Se18×M08	126	126	0			
F_2_Se18×M08	653	489	164	3:1	0.005	0.946
Se18×(Se18×M08), BC1	502	254	248	1:1	0.072	0.789

### Map-based cloning of *ClATM1*

For inheritance analyses, Se18 was used as the female parent to cross WT and M08, and all F_1_ plants were male fertile; moreover, progeny from the corresponding F_2_ and BC_1_ populations presented segregation ratios of 3:1 and 1:1, respectively (Table 1), suggesting that male sterility is controlled by a single recessive gene, which was designated *ClATM1* (*Citrullus lanatus Abnormal Tapetum 1*). Because of the low degree of genomic polymorphism between Se18 and WT, the segregating population derived from crossing Se18 with M08 was used for map-based cloning.

**Fig. 3:**
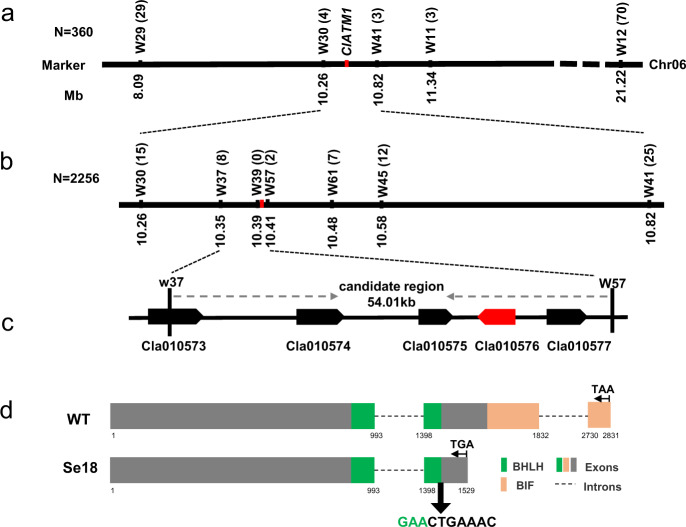
Genetic mapping of the male sterility gene *ClATM1*. **a** Primary mapping of *ClATM1*. **b** Fine mapping of *ClATM1*. The numbers in brackets after the marker name indicate the numbers of recombinants. **c** Schematic diagram of the predicted genes. Five genes were annotated within the mapping interval, and *Cla001576* (in red), which encodes a bHLH TF, was considered the most likely candidate gene. **d** *Cla010576* sequence analysis. The gray boxes, black dotted lines, green boxes, and orange boxes represent exons, introns, the bHLH domain, and the BIF domain, respectively. The black arrow indicates the 10-bp deletion of the ‘GAACTGAAAC’ nucleotide sequence, with 3 bp located at the end of the bHLH domain (the green GAA)

Using a small segregating population (*n* = 360), the *ClATM1* locus was primarily delimited into a 0.56-Mb genomic region between markers W30 and W41 on chromosome 06 (Chr06), with four and three recombinants, respectively (Fig. 3a). Then, another 40 recombinants were obtained after screening 2256 F_2_ seedlings with the markers W30 and W41. For precise mapping, five new polymorphic markers from the initial mapping interval were used to genotype these 47 recombinants. The *ClATM1* locus was further narrowed to a 54.01-kb region between the markers W37 and W57, with eight (0.35 cM) and two (0.09 cM) recombinants (Fig. 3b, Fig. S1), respectively. According to the watermelon reference annotation file, five predicted genes were identified in this 54.01-kb region (Fig. 3c). The annotations of these five predicted genes are shown in Supplementary Table S2. To identify possible candidates, we analyzed the expression patterns of these genes in male floral buds at stages 6–7, initiating cytological differences between the WT and Se18. As a result, the expression of *Cla010573*, *Cla010574*, *Cla010575*, and *Cla010577* showed slight shifts between the two parental lines, but *Cla010576* was significantly downregulated in Se18 compared with the WT (Fig. S2a). Genomic polymorphisms revealed that only the *Cla010576* gene had a 10-bp deletion in Se18, while the genomic sequences of the other four candidates were consistent between the WT and Se18 (Fig. S2b). A BLAST search showed that the Cla010576 protein was 43.64%, 42.69%, and 44.68% similar to Arabidopsis bHLH091, bHLH089, and bHLH010, respectively (Table S3, Fig. S3), all of which play essential roles in pollen and tapetum development^28^. Amino acid sequence alignment showed that a bHLH domain and a bHLH interaction and functional (BIF) domain were highly conserved among these four proteins (Fig. S3).

### *Cla010576* is preferentially expressed in the tapetum and microsporocytes

**Fig. 4:**
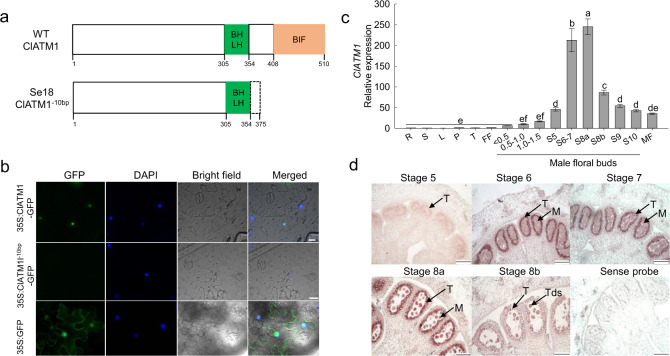
Expression pattern and subcellular localization of *Cla010576/ClATM1*. **a** The truncated forms of the Cla010576 protein in Se18. **b** Subcellular localization of Cla010576 and Cla010576^–10bp^. 35 S:Cla010576-GFP and 35 S:Cla010576^–10bp^-GFP represent Cla010576 and Cla010576^–10bp^ fused to a green fluorescence protein (GFP), respectively. GFP driven by the 35 S promoter (35 S:GFP) was used as a control. The blue points represent DAPI-stained nuclei. Scale bars, 20 μm. **c** Expression of *Cla010576* in the roots (R), stems (S), leaves (L), petioles (P), tendrils (T), female flowers (FF), different male floral buds, and open male flowers (MF) of WT plants. <0.5 (and similar values) indicates the diameter (mm) of male floral buds; S6–7 (and similar numerals) indicates the male floral buds at stages 6–7 of anther development. The expression level in the WT roots was used as a reference for relative expression. The data are presented as the means ± SDs of three replicates. The different letters indicate significant differences according to Tukey’s multiple range tests (HSD) (*P* < 0.05). **d** Expression patterns of *ClATM1* in developing WT anthers, as assessed by RNA in situ hybridization. Anthers at stage 7 hybridized to sense probe were used as negative control. M microsporocyte, T tapetum, Tds tetrads. Scale bars, 100 μm

Based on genome annotation data, *Cla010576* encodes a bHLH TF, and its 1530 bp coding DNA sequence comprises three exons (Fig. 3d). Sequencing analysis of M08, WT, and Se18 showed that there was a shared 10-bp (GAACTGAAAC) deletion in the second exon of *Cla010576* in Se18, which included 3 bp of the end of the bHLH domain (Fig. 3d, Fig. S2c). The 10-bp deletion in *ClATM1* was predicted to result in a truncated protein (375 aa, defined as Cla010576^–10bp^) without a BIF domain (Fig. 4a). Detection of GFP fluorescence signals revealed that the Cla010576 protein was localized in the nucleus, in contrast to the Cla010576^–10bp^-GFP fusion protein, which did not show any visible signals (Fig. 4b), inferring that the BIF domain is necessary for nuclear localization.

Tissue-specific expression analyses in WT plants revealed that *Cla010576* was weakly expressed in the roots, shoots, leaves, petioles, tendrils, and female flowers but highly expressed in the male floral buds, especially at stages 6–8a of anther development (Fig. 4c). Moreover, RNA in situ hybridization confirmed that *ClATM1* was initially detected in the anther tapetum at stage 5 and predominantly accumulated in the tapetum and microsporocytes during stages 6–8a (Fig. 4d), which coincided with the initial stages of defective tapetum development in Se18. On the basis of these results, the bHLH TF *Cla010576* was considered the best candidate gene for *ClATM1*.

### Mutagenesis of *Cla010576* results in a male-sterile phenotype

**Fig. 5:**
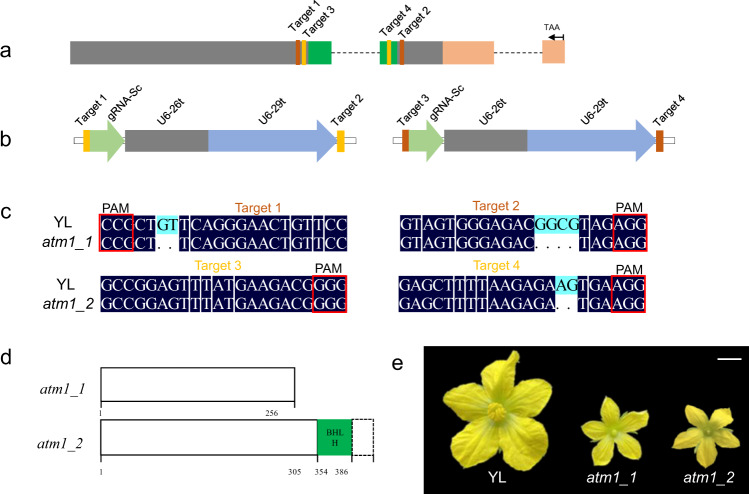
Generation and phenotypic analysis of *atm1* plants. **a** Four sgRNAs (targets 1–4) in the coding DNA sequence of *ClATM1*. **b** Schematic diagram for constructing each of the two sgRNA (target 1 and target 2; target 3 and target 4) cassettes in the binary vector PBSE402. **c** DNA sequence analysis of the targets of *ClATM1* in YL, *atm1_1*, and *atm1_2*. **d** The truncated forms of the *ClATM1* protein in *atm1_1* and *atm1_2*. **e** Phenotypes of male flowers of YL, *atm1_1*, and *atm1_2*. Scale bars, 1 cm

Considering that the Arabidopsis single mutants of the functionally redundant genes *bHLH091*, *bHLH089*, and *bHLH010* are male fertile^28^, the CRISPR/Cas9 gene-editing system was subsequently used to validate whether their orthologous gene *Cla010576* is responsible for male sterility in watermelon. Each of the two target sites in the CDS of *ClATM1* (target 1 and target 2; target 3 and target 4) was assembled into the CRISPR/Cas9 vector PBSE402 (Fig. 5a, b). After transformation of watermelon germplasm YL, two *Cla010576*-edited lines were generated, which are hereafter referred to as *atm1_1* and *atm1_2*. Line *atm1_1* had homozygous deletions of 2 bp and 4 bp in target 1 and target 2 (Fig. 5c), respectively, which generated a truncated protein (256 aa) without a bHLH or BIF domain (Fig. 5d). Line *atm1_2* had a 2-bp homozygous deletion in target 4 at the end of the bHLH domain (Fig. 5c), which led to a truncated protein (386 aa) without the BIF domain (Fig. 5d). Compared with the untransformed control line YL, *atm1_1* and *atm1_2* showed normal vegetative growth but abnormal of male flower morphology, such as smaller petals and degraded anthers with no viable pollen (Fig. 5e). Similar cytological defects in Se18 were observed in *atm1_1*, including abnormal tapetal cells, persistent callose, compressed anther locules, and degraded microsporocytes (Fig. S4). Moreover, the *ClATM1*-specific marker *Indel-Se18* based on the 10-bp deletion was 100% accurate across different populations (Fig. S5). Taken together, these results indicate that *Cla010576* (*ClATM1*) is the gene controlling male sterility in watermelon Se18.

### ClATM1 regulates its own expression through promoter binding

**Fig. 6:**
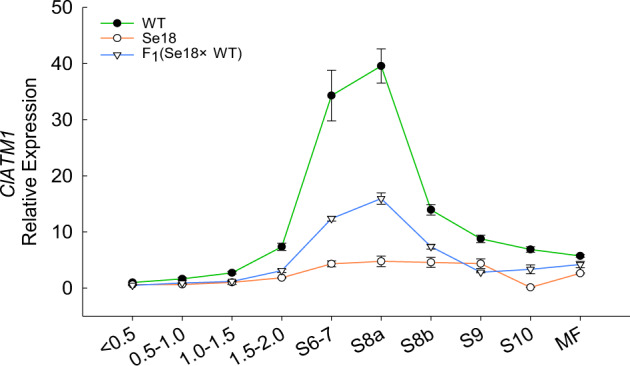
Expression of *ClATM1* in male floral buds at different developmental stages in WT plants, Se18 plants, and F1 plants. The expression level in the smallest WT buds (<0.5 mm) was used as a reference for relative expression. The <0.5 (and similar values) indicates the diameter (mm) of male floral buds; S6–7 (and similar numerals) indicates the male floral buds at stages 6–7 of anther development; MF, opening male flower. Each value is presented as the mean ± SD of three replicates

To investigate whether *ClATM1* regulates the tapetum at the transcriptional level in Se18, we compared the expression patterns of *ClATM1* in anthers of the WT and Se18 using qRT–PCR. Astonishingly, a clear dose effect of *ClATM1* was detected at the transcriptional level among the WT, Se18, and F_1_ progeny (Fig. 6). Given the dramatic reduction that occurred in Se18, the promoters of *ClATM1* and *ClATM1*^*–10bp*^ were first analyzed, revealing that there were no polymorphisms in the promoter sequence (-1 to -2028 bp) (Fig. S6). Additionally, six E-boxes (CANNTG), which are the binding sites of bHLH TFs^25,46,47^, were detected in the promoter sequences (Fig. S6). Thus, we hypothesized that the dose effect resulted from the functional ClATM1 protein, which might self-regulate its expression through promoter binding.

**Fig. 7:**
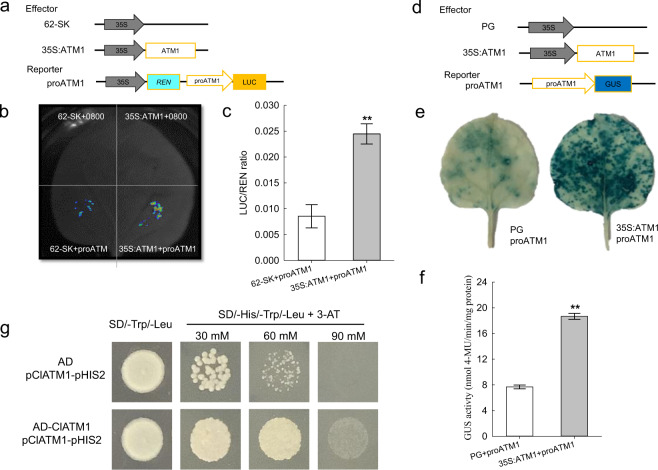
ClATM1 activates its own promoter. **a** Schematic diagrams of the effector and reporter constructs used for DLR assays. **b** LUC images of tobacco leaves after transient infiltration. 62-SK + 0800 and *35* *S:ATM1* + 0800 were used as negative controls, and 62-SK + *proATM1* was the background control. **c** The promoter activities are shown as the ratio of LUC/REN. The data are the means ± SDs of six replicates. ** represents a significant difference at *P* < 0.01 (Student’s *t* test). LUC, firefly luciferase activity; REN, *Renilla* luciferase activity. **d** Schematic diagrams of the effector and reporter constructs used for GUS analysis. **e** Image of GUS staining. PG + *proATM1* was used as a background control. **f** GUS activity. The data are shown as the means ± SDs of 12 replicates. ** represents a significant difference at *P* < 0.01 (Student’s *t* test). **g** The grow*t*h status of transformed yeast strain Y187 in SD/-Trp/-Leu and SD/-His/-Trp/-leu media with different concentrations of 3-AT (30 mM, 60 mM, or 90 mM). AD, pGADT7; pClATM1, promoter of *ClATM1*

To test this hypothesis, a DLR assay was performed after introducing the CDS and promoter of *ClATM1* into the effector and reporter vectors, respectively (Fig. 7a). As a result, much greater LUC activity was observed in the cotransformed region of the *35* *S:ATM1* effector and *proATM1* reporter vectors (Fig. 7b), with an approximately 2.86-fold higher LUC/REN ratio than that in the empty (62-SK) effector group (Fig. 7c). Similarly, the β-glucuronidase (GUS) assay also revealed the physical interaction between the ClATM1 and its promoter (Fig. 7d), as active GUS expression was significantly enhanced (approximately 2.43-fold) in *35* *S:ATM1* cells compared with empty vector-transformed control cells (Fig. 7e, f). To further validate this interaction, a Y1H assay was conducted, and the interaction between ClATM1 and its promoter was also confirmed (Fig. 7g). Overall, these findings indicated that ClATM1 could bind to its own promoter to activate its own transcriptional activity, confirming the dose effect.

## Discussion

Watermelon is an economically important cucurbit crop species worldwide. In addition, male sterility has been used for hybrid seed production to increase crop yields and improve disease resistance^2,48^. However, male sterility has not been extensively applied for the utilization of hybrid vigor in watermelon because no male sterility genes have been identified in this species. In this study, we identified the first male sterility gene, *ClATM1*, in watermelon and revealed the self-regulatory role of *ClATM1* in tapetum development.

### *ClATM1* regulates tapetum development in watermelon

Proper development of sporophytic cell layers, including the tapetum, is essential for successful pollen development in plants^23,49^. Our data showed that Se18 and *atm1_1* mutants underwent normal cell division to generate four anther wall layers at stage 5 (Fig. 2k, S4a); the defective tapetum was initially detected following its formation at stages 6–7 (Fig. 2). In addition, *ClATM1* was expressed preferentially in the tapetum and microsporocytes during stages 6–8a of anther development (Fig. 4c, d), which coincided with the initial stages in which phenotypic defects were observed, demonstrating the spatiotemporal functions of *ClATM1*. The tapetum secretes β-1,3-glucanase in the locule to degrade the callose wall surrounding tetrads to release microspores^45^. The callose wall was continuously present in both Se18 and the transgenic line *atm1_1* (Fig. 2z–ab, S4h–j), further confirming their abnormal tapetum development. Therefore, we demonstrated that *ClATM1* is required for tapetum and anther development in watermelon.

The features concerning the generation, development, and apoptosis of the tapetum are diverse^10,13,42,43^. The tapetum defects of the *atm1* mutants were compared with those of other orthologous mutants of other plant species (Fig. S7). Unlike these expanded tapetal cells that occur in Arabidopsis *bhlh091/bhlh089/bhlh010*^28^, rice *tip2*^27^, tomato *ms32*^50^, and Medicago *ean1–1*^17^ mutants, the thicker tapetal cells that occur in the rice *eat1–1* mutant^3^, or the double-layer tapetal cells that occur in maize *ms23*^22^, the tapetal cells of Se18 and *atm1_1* proliferated extensively, were multilayered, smaller in size, irregular in shape, and not radially elongated in the early stages (Figs. 2l–t, S4c–f). Overall, we speculate that *ClATM1* plays both key and unique regulatory roles in tapetum and anther development.

### *ClATM1* is a single-copy gene

In plants, the bHLH gene family can be further divided into at least 26 well-defined subfamilies, some of which are highly conserved and associated with the tapetum, such as members of subfamily II (e.g., Arabidopsis *bHLH010/bHLH089/bHLH090*^28^, rice *EAT1/TIP2*^3,26,27^, maize *MS23*^22^, tomato *MS32*^50^, and Medicago *EAN1*^17^) and subfamily III(a + c)1 (e.g., Arabidopsis *AMS*^20^ and *DYT1*^18^, tomato *MS10*^*35*^ ^23^, rice *TDR*^12^ and *UDT1*^21^, and maize *MS32*^51^). In this study, the bHLH TF-encoding gene *ClATM1* was clustered together with its orthologs to form subfamily II (Fig. S7), which included Arabidopsis *bHLH00/bHLH089/bHLH091*, rice *EAT1/TIP2*, maize *bHLH122/MS23*, tomato *MS32/Solyc01g081090*, and Medicago *EAN1/EAN2/EAN3*. In rice, two functionally distinctive paralogs, *EAT1* and *TIP2*, play essential roles in tapetum degeneration; single mutations of these genes result in complete male sterility^3,27^. In maize, *bHLH122* and *MS23* are also defined as two independent monocot-specific clades, and the *ms23* mutant is completely male sterile^22^. In tomato and Medicago, single mutations in *MS32* or *EAN1* induce abnormal tapetum development and lead to complete male sterility^17,50^, whereas only the Arabidopsis triple mutant *bHLH10/bHLH89/bHLH91* is completely male sterile, suggesting these genes have redundant functions^28^. It is worth noting that at least two members were identified in subfamily II in other species, while only one gene, *ClATM1*, was detected in watermelon as well as in other Cucurbitaceae species, such as cucumber (*Cucumis sativus* L., *CsaV3_2G011050.1*) and melon (*Cucumis melo* L., *MELO3C020370*) (Fig. S7), indicating the possibly diverse mechanisms of bHLH subfamily II genes involved in tapetum development in plants. More importantly, the functions of *ClATM1* homologs and other male sterility-regulating genes have not been reported in Cucurbitaceae species. Therefore, our study provides insight into the possible roles of these TFs in Cucurbitaceae and other crop species.

### The self-regulation of *ClATM1* plays key roles in watermelon tapetum development

Previous studies have suggested that the BIF domain is required for AtDYT1 dimerization, nuclear localization, and transcriptional activation activity in Arabidopsis^52^. Our results showed that the BIF-free protein ClATM1^–10bp^ had no nuclear localization signal (Fig. 4b), suggesting that the BIF domain is necessary for ClATM1 nuclear localization. Considering that the BIF domain is required for functional ClATM1, it was reasonable to propose that the BIF-free protein ClATM1^–10bp^ may lose the ability to transcriptionally activate its downstream targets for regulating tapetum development.

In tomato, polymorphic mutations in the CDS of *SlMS23* lead to male sterility, but SlMS23 expression is not altered in the *ms23* mutant^50^. However, an obvious dose effect was detected at the transcriptional level among the three *ClATM1* genotypes (Fig. 6). Therefore, it was hypothesized that functional ClATM1 can self-regulate its own expression via promoter binding, as verified by DLR, GUS, and Y1H assays (Fig. 7). When functional ClATM1 is disrupted in *atm1* mutants, its expression levels and that of other downstream genes may be altered, resulting in defective tapetum development. In general, we demonstrated that *ClATM1* is a regulator of self-regulatory mechanisms, providing new insights into the regulation of bHLH TFs involved in male reproductive development.

## Conclusion

In summary, we demonstrated that *ClATM1* acts as a regulator of tapetum and microspore development in watermelon. On the one hand, *ClATM1* is the first male-sterility gene to undergo map-based cloning and to be functionally verified in cucurbit crop species. This gene can be greatly exploited to benefit the utilization of male sterility in cucurbit crop species. Moreover, the self-regulatory ability of *ClATM1* provides new insights into the roles of bHLH proteins in plant reproduction. These findings will aid in the marker-assisted selection of male sterility in watermelon hybrid breeding programs.

## Materials and methods

### Plant materials

The male-sterile mutant Se18 was naturally derived from watermelon (*Citrullus lanatus* L.) ‘Sugarlee’, and its homozygous male-fertile line was considered the wild type (WT). The inbred line M08 was the male parent, which was crossed with Se18 to generate an F_2_ mapping population. Additionally, the BC_1_ and F_2_ populations resulting from the crosses of Se18 × WT and Se18 × M08 and 30 additional watermelon lines were used to analyze the inheritance and validate the presence of the *Indel-Se18* marker. All plant materials, including *atm1* mutants and YL, were grown at the farms of Northwest A&F University, Yangling, Shaanxi, China.

### Phenotypic analysis and microscopy examination

Images of plants and male flowers were taken with a Nikon DXM1200 digital camera. Both pollen viability and paraffin sections were respectively determined and prepared as described in previous studies^53,54^. For callose observations, the same sections mentioned above were stained with 0.01% aniline blue^55^. All the observations were conducted with the aid of an Olympus BX63 microscope (Tokyo, Japan).

### Molecular marker development and fine gene mapping

After comparing the genome resequencing data of Se18 and M08, single-nucleotide polymorphism variations were assessed to develop corresponding cleaved amplified polymorphic sequence (CAPS) markers. For preliminary mapping, 22 CAPS markers evenly distributed across all 11 chromosomes were used for linkage analysis with F_2_ recessive individuals. Next, three new markers were used to screen a smaller F_2_ segregating population (*n* = 360) to delimit the primary target interval. For fine mapping, the whole F_2_ population (*n* = 2256) was genotyped, with W30 and W41 used. A total of 47 recombinants were selected for further genotyping involving five new markers. The information concerning the markers used is listed in Table S4.

### Gene prediction and sequence polymorphism analysis

The putative genes within the mapping intervals were identified on the basis of the information within the watermelon database 97103 V1 (http://cucurbitgenomics.org/organism/1). The genomic and coding DNA sequences of the candidate genes were independently amplified from Se18, WT, and M08 by gene-specific primers (Table S5) and subsequently sequenced by Tsingke Biotech (Beijing, China).

### Subcellular localization

The coding DNA sequences (CDSs) of *Cla010576* and *Cla010576*^*–10bp*^ without the stop codon were independently amplified and inserted into the plasmid pGreenII-35S-GFP. The constructs were then transiently transformed into tobacco (*Nicotiana benthamiana*) leaves through *Agrobacterium tumefaciens* infiltration^56^. After 48 h, the epidermis of the tobacco leaves was injected with DAPI and examined with an Olympus BX63 fluorescence microscope (Tokyo, Japan). All the primers used in this experiment are listed in Table S5.

### Quantitative real-time PCR (qRT–PCR) assays

Different tissues and male floral buds at different developmental stages were collected from Se18 and WT plants and flash frozen in liquid nitrogen. Total RNA was isolated using an RNA Simple Total RNA Kit (DP432, Tiangen, China), and cDNA was synthesized using a Fast King RT Kit in conjunction with gDNase (KR116, Tiangen, China). SYBR Green PCR Master Mix (Applied Biosystems^®^, Inc., Foster, USA) and a Step-One Plus Real-Time PCR system (Applied Biosystems^®^) were used to conduct qRT–PCR. The housekeeping gene *Cla007792* was used as an internal reference^57^. The relative mRNA expression was determined using the 2^-∆∆CT^ method^58^. All the gene-specific primers used for qRT–PCR are listed in Table S5.

### RNA in situ hybridization

Male floral buds of different sizes from the WT were fixed in FAA solution for 16 h at 4 °C. After dehydration and embedding, the anthers were sectioned to a thickness of 10 μm using an RM2245 rotary microtome (Leica, Wetzlar, Germany). Probes for *ClATM1* were designed to target a nonconserved CDS region. The sense and antisense probes were synthesized using SP6 and T7 polymerase with RNA polymerase, and a DIG RNA Labeling Kit (Roche, Rotkreuz, Switzerland) was used. RNA hybridization and immunological detection were performed as described previously^59,60^. Images were obtained using an Olympus BX63 fluorescence microscope (Tokyo, Japan).

### Vector construction and watermelon transformation

To edit *Cla010576* with the CRISPR/Cas9 system, targets were designed and selected using CRISPR-P V2.0^61^. The guide RNA sequences were constructed and inserted into the CRISPR/Cas9 vector pBSE402^62,63^. *Agrobacterium tumefaciens* strain EHA105 harboring the recombinant vector pBSE402-gRNA was used for watermelon transformation according to a previously described method^64^. Because of the low transgenic efficiency of M08 and the WT, we used the watermelon male-fertile inbred line YL as a transgenic material. Genomic DNA was extracted from YL and the stable transgenic lines, and the potential edited fragments of *Cla010576* were amplified and sequenced to identify positive transformed plants.

### Marker development

An *Indel-Se18* marker was developed based on the nucleotide variation in *ClATM1* between Se18 and WT. The PCR products were separated by electrophoresis in 8% polyacrylamide gels and subsequently visualized with silver staining.

### Dual-luciferase reporter (DLR) assays

DLR assays were conducted as described previously^65^. The full-length CDS of *ClATM1* was amplified and inserted into pGreenII 62-SK as an effector, and its promoter (approximately -1 to -2028) was inserted into pGreen II 0800-LUC as a reporter. The effector and reporter constructs were transformed into GV3101-pSoup and then transiently cotransformed (effector:reporter = 9:1) into tobacco leaves. For each TF-promoter interaction, three independent experiments with three replicates were performed. Firefly luciferase (LUC) and *Renilla* luciferase (REN) activities were assayed by a Dual-Luciferase Reporter Gene Assay Kit (Yeasen, Shanghai, China) with an Infinite M200 Pro Microplate Reader (Tecan, Switzerland).

### GUS activity analysis

The CDS and promoter of *ClATM1* were amplified and ligated into the effector vector pGreenII-35S-GFP and the reporter vector pBI121-GUS, respectively. The constructs were induced to *Agrobacterium tumefaciens* (strain GV3101-pSoup), which was then transiently cotransformed into the tobacco leaves. After 48 h, the leaves were immersed in staining buffer at 37 °C for 24 h and then rinsed with 70% (v/v) ethanol^66^. The other leaves were frozen in liquid nitrogen to analyze GUS activity according to a previously described method^67^.

### Yeast one-hybrid (Y1H) assays

Y1H assays were conducted using a Matchmaker^TM^ Gold Yeast One-Hybrid Library Screening System Kit (cat. No. 630491, Clontech, CA, USA)^66^. The full-length CDS and approximately 2028-bp promoter sequence of *ClATM1* were inserted into pGADT7 (AD) and pHIS2, respectively. AD/pATM1-pHIS2 and AD-ATM1/pATM1-pHIS2 were cotransformed into the Y187 strain and cultured on SD/-Trp/-Leu for 2–3 days at 30 °C. Then, healthy and consistent clones were diluted, and 2 μL was added to SD/-Trp/-Leu and SD/-His/-Trp/-Leu media with different concentrations of 3-amino-1,2,4-triazole (3-AT) (30 mM, 60 mM or 90 mM) to detect protein interactions.

### Phylogenetic tree construction

The protein sequences of ClATM1 and its orthologs were obtained from the Cucurbit Genomics Database (http://cucurbitgenomics.org/) and from the data of previous studies^17^. Multiple sequence alignment was conducted using MUSCLE. A neighbor-joining tree was generated via 1000 bootstrap replicates using MEGA 7.0^57,68^.

## Supplementary information

Supporting information R1-Clean version

## Data Availability

All the relevant data can be found within this manuscript and its supporting information files.
