# Supplementary material for: Disruption of the bHLH transcription factor Abnormal Tapetum 1 causes male sterility in watermelon
Source: Hortic Res. 2021 Dec 1;8:258. doi: 10.1038/s41438-021-00695-9 (PMC8632879; doi:10.1038/s41438-021-00695-9)
Supplement: Supplementary file 1 — Supporting information R1-Clean version [file 41438_2021_695_MOESM1_ESM.docx]

**SUPPORTING INFORMATION**

**Fig. S1 Marker genotypes of the recombinants between W37 and W57.** MS, Male Sterility; MF, Male Fertility; B, Recessive Homozygous; H, Heterozygous.

**Fig. S2 Predicted candidate genes.** (a). Expression analysis of candidate genes in male floral buds of stages 6-7 in WT and Se18. Data was represented as means ± SD of three replicates. (b) Nucleotide polymorphisms of candidate genes between WT and Se18. (c) Comparison of genomics sequence of *Cla010576* in 97103, WT, Se18 and M08.

**Fig. S3** **Comparison of the Cl010576 protein sequence with the AtbHLH091, AtbHLH010 and AtbHLH089 sequences.** The bHLH domain and BIF domain were indicated by green and orange square, respectively.

**
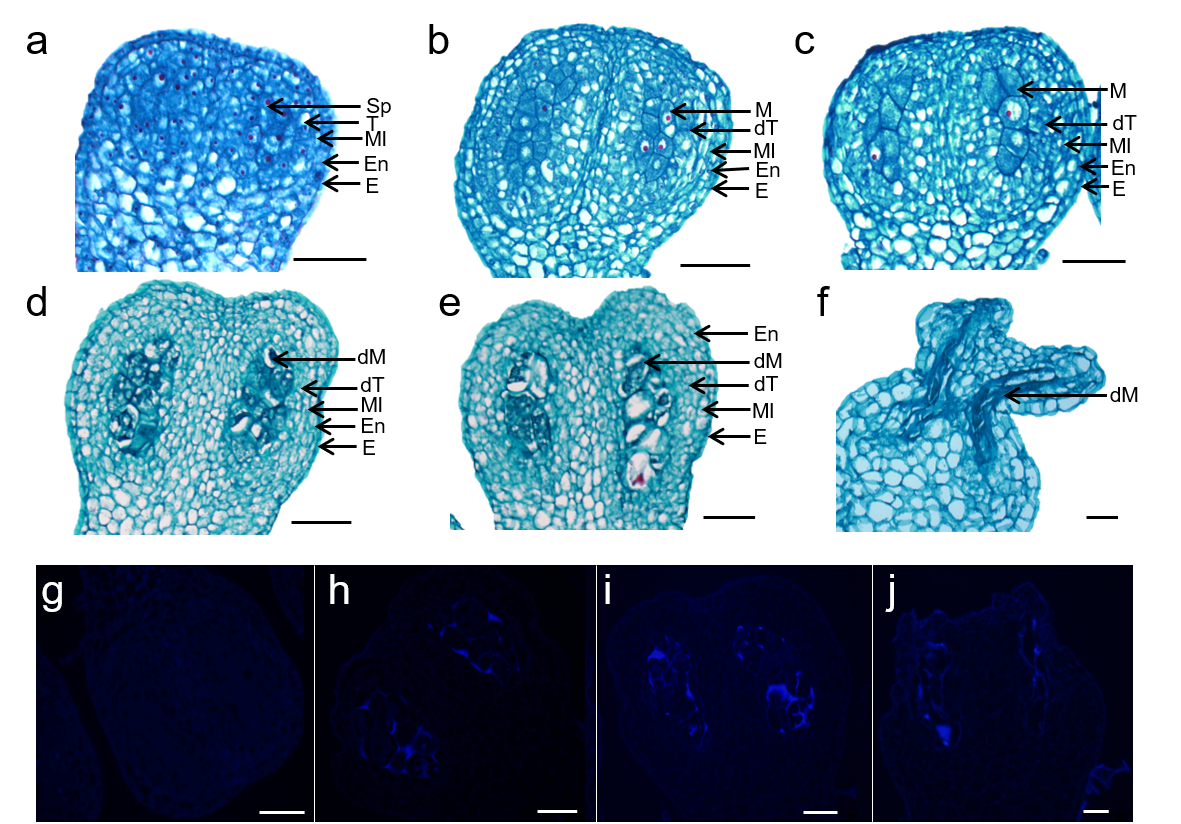
**

**Fig. S4 Transverse sections of atm1-1 anther of different sizes.** The corresponding floral bud diameter sizes (mm) are presented in Table S1. dM, defective Microsporocyte; dT, defective Tapetum; E, Epidermis; En, Endothecium; M, Microsporocyte; Ml, Middle layer; Sc, Sporogenous cell; T, tapetum; Scale bars, 50 μm. (d) Callose analysis in *atm1-1* anthers of different sizes. Scale bars, 50 μm.

**Fig. S5 Polymorphism of the molecular marker Indel_Se18 in different watermelon materials. (**a) 1: WT, 2: Se18, 3: F_1_ Se18 × WT, 4-13: F_2_ Se18 × WT, Sterile plants, 10, 14-43：F_2_ Se18 × WT, Fertile plants, 30. (b) 1: WT, 2: Se18, 3: F_1_ Se18 × WT, 4-24: Se18 × (Se18 × WT), BC_1_, Sterile plants, 20, 25-43: Se18 × (Se18 × WT), BC_1_, Fertile plants, 20. (c) 1: M08, 2: Se18, 3: F_1_ Se18 × M08, 4: WT, 5: Se18, 6: F_1_ Se18 × WT, 7-37: different fertile materials, 30.

**Fig. S6** **Comparison of ClATM1 promoter sequences among 97103, WT and Se18.** The E-box (CANNTG) was marked in the promoter (-1 to -2028) of *ClATM1*.

**Fig. S7 Phylogenetic analysis of ClATM1 and its orthologs.** *At, Arabidopsis thaliana; Cm, Cucumis melo; Cl, Citrullus lanatus; Cs, Cucumis sativus; Mt, Medicago truncatula; Os, Oryza sativa; Sl, Solanum lycopersicum; Zm, Zea mays.*

**Table S1 Transverse diameters of watermelon floral buds at different developmental stages and of different sizes used in this study.**

| **Anther stages** | **Floral buds diameter size (mm)** | | | | **Floral buds sizes used in Fig. 2 (mm)** | | | **Floral buds sizes used in Fig. S4 (mm)** |
| --- | --- | --- | --- | --- | --- | --- | --- | --- |
|  | **Y2018** | **Y2019** | **Y2020** | **Inference** | **Wild Type** | **Se18** | **u-ab** |  |
| Stage 5 | 1.76 | / | 1.81 | 1.5~2.0 | 1.76*2.33 (a) | 1.76*1.90 (k) | **Wild Type** | 1.50*2.08 (a) |
| Stage 6 | / | 1.88 | 2.13 | 2.0~2.5 | 2.13*2.40 (b) | 2.00*2.30 (l) | 2.74*3.02 (u) | 2.15*2.30 (b) |
| Stage 7 | 2.24 | 2.55 | 2.34 |  | 2.34*2.95 (c) | 2.49*3.20 (m) | 3.28*3.25(v) | 2.45*3.20 (c) |
| Stage 8a | 2.6 | 2.74 | 2.85 | 2.5~3.0 | 2.74*3.02 (d) | 2.97*3.62 (n) | 4.27*4.71(w) | 3.00*4.13 (d) |
| Stage 8b | 3.12 | 3.28 | / | 3.0~3.5 | 3.28*3.25 (e) | 3.41*3.89 (o) | 7.57*11.94(x) | 3.32*5.59 (e) |
| Stage 9 | 3.61 | / | 3.71 | 3.5~4.0 | 3.71*4.20 (f) | 3.52*4.10 (p) | **Se18** | Flowering (f) |
| Stage 10 | 4.24 | 4.27 | 4.41 | 4.0~4.5 | 4.27*4.13 (g) | 4.67*6.87 (q) | 2.49*2.17(y) | 1.50*2.08 (h) |
| Stage 11 | 5.72 | 5.56 | 5.39 | 5.0~6.0 | 5.39*6.08 (h) | 5.08*7.92 (r) | 3.19*3.34(z) | 2.15*2.30 (i) |
| Stage 12 | 1day before flowering | | | | 1day before flowering (i) | 1day before flowering (s) | 3.40*4.27(aa) | 2.60*3.08 (j) |
| Stage 14 | Flowering | | | | Flowering (j) | Flowering (t) | 4.03*4.55(ab) | 3.32*5.59 (k) |

**Table S2 Predicted genes between markers W37 and W57.**

|  | **Gene ID** | **Position** | **CDS (bp)** | **NCBI BlastP Hit** |
| --- | --- | --- | --- | --- |
| Chr06 10359145-10413150 | Cla010573 | 10358577–10360277 | 753 | putative casein kinase II subunit beta-4 |
|  | Cla010574 | 10377096–10379032 | 720 | phosphatidylinositol 4-phosphate 5-kinase 9-like |
|  | Cla010575 | 10385775–10386656 | 276 | 60S ribosomal protein L37a-1-like |
|  | Cla010576 | 10388277–10391107 | 1530 | transcription factor bHLH91-like |
|  | Cla010577 | 10394586–10394858 | 273 | uncharacterized protein |

**Table S3 Homology among Cl010576, AtbHLH091, AtbHLH010 and AtbHLH089.**

| **Watermelon** | | ***Arabidopsis thaliana*** | | **Identity** | **E-value** | **Description** |
| --- | --- | --- | --- | --- | --- | --- |
| **Gene name** | **Gene ID** | **Gene name** | **Gene ID** |  |  |  |
| *ClATM1* | *Cla010576* | *AtbHLH091* | *AT2G31210* | 43.64% | 7.6E-70 | bHLH transcription factor |
|  |  | *AtbHLH089* | *AT1G06170* | 42.69% | 3E-58 |  |
|  |  | *AtbHLH010* | *AT2G31220* | 44.68% | 3.8E-69 |  |

**Table S4 Information concerning the markers used for gene mapping.**

| **Makers** | **Primer Sequence** | **SNP position** | **Restriction enzyme** | **Product size** |
| --- | --- | --- | --- | --- |
| W11 | ACCTAGTGTCTAGCGTACGGAAGTT | Chr06_11409496 | *Hha*I | 374 |
|  | TCCGTTAACTGCTGGTTGGCTGT |  |  |  |
| W12 | TGCAATGCCAGGGAGGGGTAGTT | Chr06_21220014 | *Dra*I | 592 |
|  | TGACTCAACCATGACAGGTGGTT |  |  |  |
| W29 | ACAGCCCAAGGGCAGAGGGT | Chr06_8091722 | *Hinf*I | 485 |
|  | TGTGGTTGGAGACCAAAGTGACA |  |  |  |
| W30 | TGGGGTGTGTGCTGTGTTGTGG | Chr06_10267230 | *Pvu*II | 726 |
|  | AATGCCCCTCATGGCTGCCG |  |  |  |
| W37 | AGGTCATTTAATGTCTCTGTTCCGTGT | Chr06_10359145 | *Dra*I | 521 |
|  | GTCAAGGAAGATTATGAGGGCAGCA |  |  |  |
| W39 | TCTGGAACTCGCCGCCGGTA | Chr06_10390616 | *Ava*II | 538 |
|  | CTCCGGCGCTGACGCTATGG |  |  |  |
| W45 | GGAGGCGAGGAACTGCGCTC | Chr06_10581524 | *Dra*I | 364 |
|  | TCTAGTCTATCACTAACTATCACGGA |  |  |  |
| W41 | TGCGCTTCTTCACCAGGGGC | Chr06_10828069 | *Hinf*I | 695 |
|  | TGAGTGGCTTGGCCCCCAAA |  |  |  |
| W51 | AATTCCTTAACTGGGTTGAACTGCAT | Chr06_10554492 | sequence testing | 545 |
|  | GCATTGCAGCTGGCTCTCCCT |  |  |  |
| W57 | CTTGGTGCAAACCCCAACAA | Chr06_10413150 | *Hpa*II | 709 |
|  | TCGTACTACCTGTTGGGACCT |  |  |  |

**Table S5 Primers used in this study.**

| **Experiment** | **Primer name** | **Primer Sequence (5'-3')** |
| --- | --- | --- |
| For Sequenceing | Cla010576 F/R | ATGTATGAACAAACTGGGTGTTCTG / TTAATAGGTATTGGATGTGGTGGTG |
| For Indel maker | Indel_Se18 F/R | GGGCATCAGTTGTGGGAGACGC / CCGCCGTCCTGTGCCTCTTG |
| For qRT-PCR | Cla007792 F/R | CCATGTATGTTGCCATCCAG / GGATAGCATGGGGTAGAGCA |
|  | ClATM1 qF/qR | CCATGCCCACTGCCTATACT / AAGCGAAGTCGACGATGAGT |
| For Subcellular location | 35S: ClATM1_GFP_F | GAGGACAGCCCAAGCTACGCGTCTCGAGATGTATGAACAAACTGGGTGTTCTG |
|  | 35S: ClATM1_GFP_R | ATCCCCCGGGCTGCAGGAATTCGATATCATAGGTATTGGATGTGGTGGTG |
|  | 35S: ClATM1^-10bp^_GFP_R | ATCCCCCGGGCTGCAGGAATTCGATATCATTCCTCCGCCGTCCTGTGC |
| For gene editing | ClATM1_CRguide1_F | ATATATGGTCTCGATTGGGAACAGTTCCCTGAACAGGTTTTAGAGCTAGAAATAGC |
|  | ClATM1_CRguide2_R | ATTATTGGTCTCGAAACCTACGCCGTCTCCCACTACCAATCTCTTAGTCGACTCTAC |
|  | ClATM1_CRguide3_F | TCGAAGTAGTGATTGGCCGGAGTTTATGAAGACGGTTTTAGAGCTAGAAATAGC |
|  | ClATM1_CRguide4_R | TTCTAGCTCTAAAACTCACTTCTCTTAAAAGCTCCAATCTCTTAGTCGACTCTAC |
| For DLR | 62-SK-ATM1 F | CGCTCTAGAACTAGTGGATCCATGTATGAACAAACTGGGTGTTCTG |
|  | 62-SK-ATM1 R | CGATAAGCTTGATATCGAATTCTTAATAGGTATTGGATGTGGTGGTG |
|  | 0800LUC-pATM1 F | GGTATCGATAAGCTTGATATCTATTATCAGGGGTCTTTGTCTTAG |
|  | 0800LUC-pATM1 R | CGCTCTAGAACTAGTGGATCCATTTTGGTAAGAAAAGTGTGCAGCT |
| For GUS assay | PBI121-GUS-pATM F | GAGAACACGGGGGACTCTAGATATTATCAGGGGTCTTTGTCTTAG |
|  | PBI121-GUS-pATM R | GGACTGACCACCCGGGGATCCATTTTGGTAAGAAAAGTGTGCAGCT |
|  | 35S: ClATM1_GFP_F | GAGGACAGCCCAAGCTACGCGTCTCGAGATGTATGAACAAACTGGGTGTTCTG |
|  | 35S: ClATM1_GFP_R | ATCCCCCGGGCTGCAGGAATTCGATATCATAGGTATTGGATGTGGTGGTG |
| For Y1H | pGADT7_ClATM1 F | GCCATGGAGGCCAGTGAATTCATGTATGAACAAACTGGGTGTTCTG |
|  | pGADT7_ClATM1 R | CAGCTCGAGCTCGATGGATCCTTAATAGGTATTGGATGTGGTGGTG |
|  | pHIS2-pATM1-F | GACTCACTATAGGGCGAATTCTATTATCAGGGGTCTTTGTCTTAG |
|  | pHIS-pATM1-R | GATTCGCGAACGCGTGAGCTCATTTTGGTAAGAAAAGTGTGCAGCT |
| For situ hybridization | SP6-ClATM1 | GATTTAGGTGACACTATAGAATGCTGGTGTTCTGATCCCGATTCCA |
|  | T7-ClATM1 | TGTAATACGACTCACTATAGGGGGTGAGGTATGGTGATTGGAG |
|  | SP6-ClAMSL | GATTTAGGTGACACTATAGAATGCTCCTGATGGAGAGACTAAGAC |
|  | T7-ClAMSL | TGTAATACGACTCACTATAGGGGGCTTTGATTGGATTCCGTTTGT |
|  | SP6-ClDYT1L | GATTTAGGTGACACTATAGAATGCTGGAGCTCTGGAGTGTCTTAGA |
|  | T7-ClDYT1L | TGTAATACGACTCACTATAGGGGTATTGATGCTCATTGGAAGGC |
